# Primary large cell neuroendocrine carcinoma of the breast diagnosed using dynamic contrast-enhanced magnetic resonance imaging and diffusion-weighted imaging: A case report

**DOI:** 10.1097/MD.0000000000046558

**Published:** 2025-12-19

**Authors:** Boram Kim, Seoyun Choi, Kyoung Min Kim, Eun Jung Choi

**Affiliations:** a Department of Radiology, Chonbuk National University Hospital, Jeonju, Republic of Korea; b Department of Radiology and Research Institute of Clinical Medicine, Jeonbuk National University-Biomedical Research Institute, Jeonbuk National University Hospital, Jeonbuk National University Medical School, Jeonju, Republic of Korea; c Departments of Pathology, Jeonbuk National University Medical School, Research Institute of Clinical Medicine, Jeonbuk National University, Biomedical Research Institute of Jeonbuk National University Hospital, Research Institute for Endocrine Sciences, Jeonju, Republic of Korea; d Department of Radiology and Research Institute of Clinical Medicine, Jeonbuk National University-Biomedical Research Institute, Jeonbuk National University Hospital, Jeonbuk National University Medical School, Jeonju, Republic of Korea.

**Keywords:** breast cancer, diffusion-weighted image, dynamic contrast-enhanced magnetic resonance imaging, large cell neuroendocrine carcinoma, peritumoral-to-tumoral ADC ratio

## Abstract

**Rationale:**

Large cell neuroendocrine carcinoma of the breast (LCNECB) is extremely rare, accounting for <0.1% of breast cancers, and has been associated with aggressive clinical behavior. Owing to its rarity, diagnostic and prognostic imaging features remain poorly defined.

**Patient concerns:**

A 55-year-old woman was referred for evaluation after an abnormality was detected on screening mammography. She had no personal or family history of breast cancer.

**Diagnoses:**

Mammography and ultrasound revealed a left breast mass with irregular margins. Breast magnetic resonance imaging demonstrated rim enhancement, adjacent vessel sign, increased ipsilateral vascularity, and restricted diffusion. Diffusion-weighted imaging showed an apparent diffusion coefficient (ADC) of 0.745 × 10^−3^ mm^2^/s, with a peritumoral-to-tumoral ADC ratio of 2.79. Histopathology and immunohistochemistry confirmed LCNECB.

**Interventions:**

The patient underwent a partial mastectomy and sentinel lymph node biopsy following preoperative imaging evaluation.

**Outcomes:**

The tumor measured 3.1 cm, with lymphatic invasion but no nodal metastasis. The patient did not return after surgery and was lost to follow-up; therefore, subsequent clinical outcomes could not be assessed.

**Lessons:**

This case highlights the diagnostic potential of breast magnetic resonance imaging in LCNECB, particularly the presence of pronounced hypervascularity and a markedly elevated peritumoral-to-tumoral ADC ratio, which may serve as valuable imaging markers for prognosis and differentiation.

## 1. Introduction

Neuroendocrine carcinoma of the breast (NECB) is a distinct malignant tumor that demonstrates neuroendocrine differentiation based on histopathological and immunohistochemical findings. Neuroendocrine carcinomas usually occur in the lungs or gastrointestinal tract, and their development in the breasts is extremely rare. According to the 2022 WHO classification, primary breast neuroendocrine tumors (NETs) are classified as a new type of pathological breast cancer, which is different from invasive breast cancer with neuroendocrine differentiation. It is currently classified as a well-differentiated NET (carcinoid-like and atypical carcinoid-like) and a poorly differentiated NET (small cell neuroendocrine carcinoma and large cell neuroendocrine carcinoma). NECB accounts for 2% to 5% of breast cancers, whereas large-cell NETs account for <0.1% of all breast cancers.^[1]^ Owing to its rarity, research on the diagnosis and prognosis of large cell neuroendocrine carcinoma of the breast (LCNECB) is still limited. Histopathological findings, such as cytological features or neuroendocrine markers such as neuron-specific enolase, chromogranin A, synaptophysin, and insulinoma-associated protein 1, are known to support the diagnosis of LCNECB. However, as a noninvasive alternative to pathological confirmation following excisional or core-needle biopsy, breast imaging plays a key role in the early detection and differential diagnosis of LCNECB.

## 2. Case report

**Figure 1.**
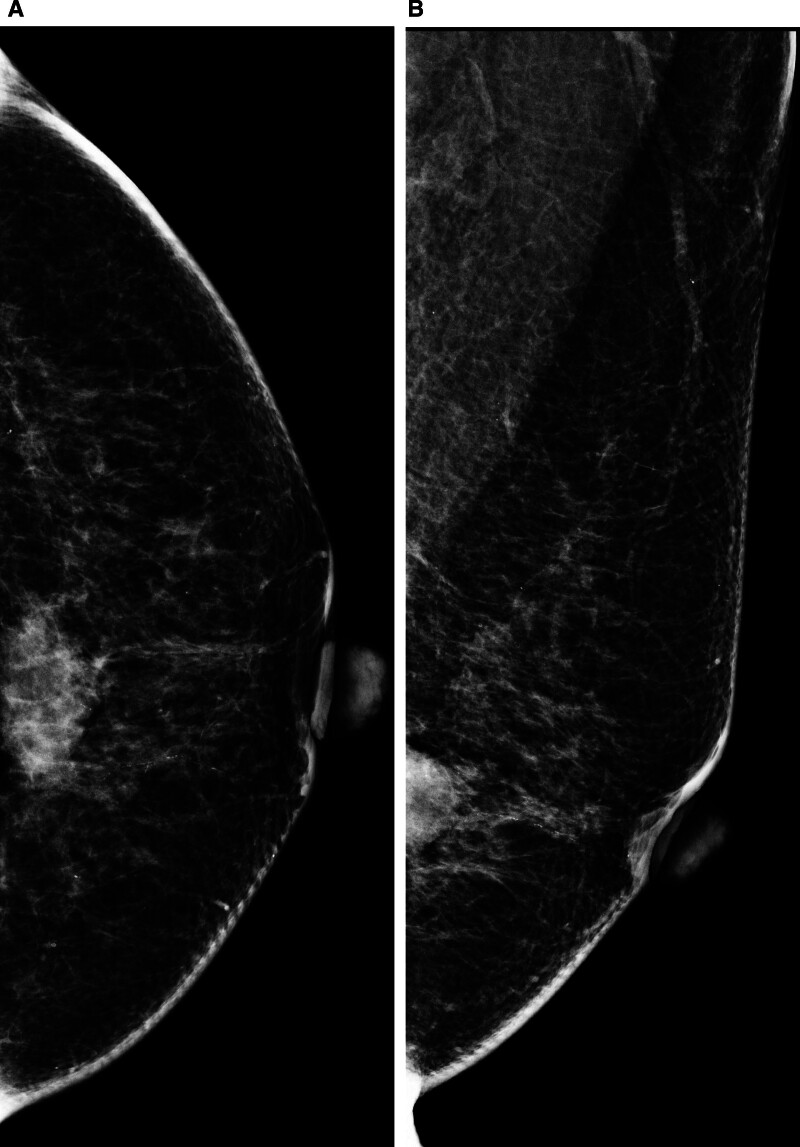
Mammographic findings of large cell neuroendocrine carcinoma. Bilateral craniocaudal (CC) (A) and mediolateral oblique (MLO) (B) views showed a 3-cm hyperdense mass in the lower central quadrant of the left breast, with an irregular shape and microlobulated margins, without microcalcifications or associated skin changes. CC = craniocaudal, MLO = mediolateral oblique.

**Figure 2.**
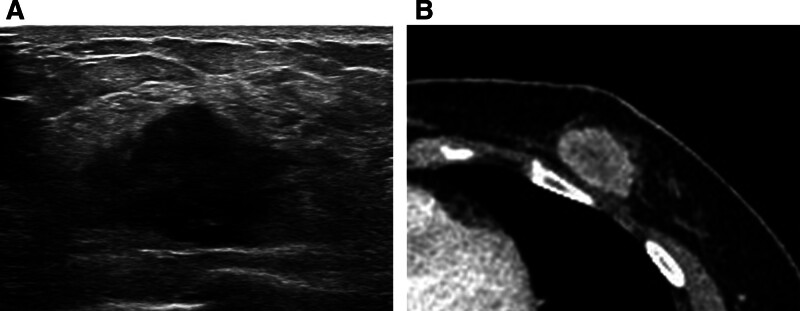
Breast ultrasound and CT findings of large cell neuroendocrine carcinoma. (A) The breast ultrasound image showed a hypoechoic mass with an irregular shape and microlobulated margins at the 6 o’clock position of the left breast, without posterior acoustic features or associated findings. (B) Chest CT revealed a rim-enhancing mass with a microlobulated margin in the left breast. CT = computed tomography.

**Figure 3.**
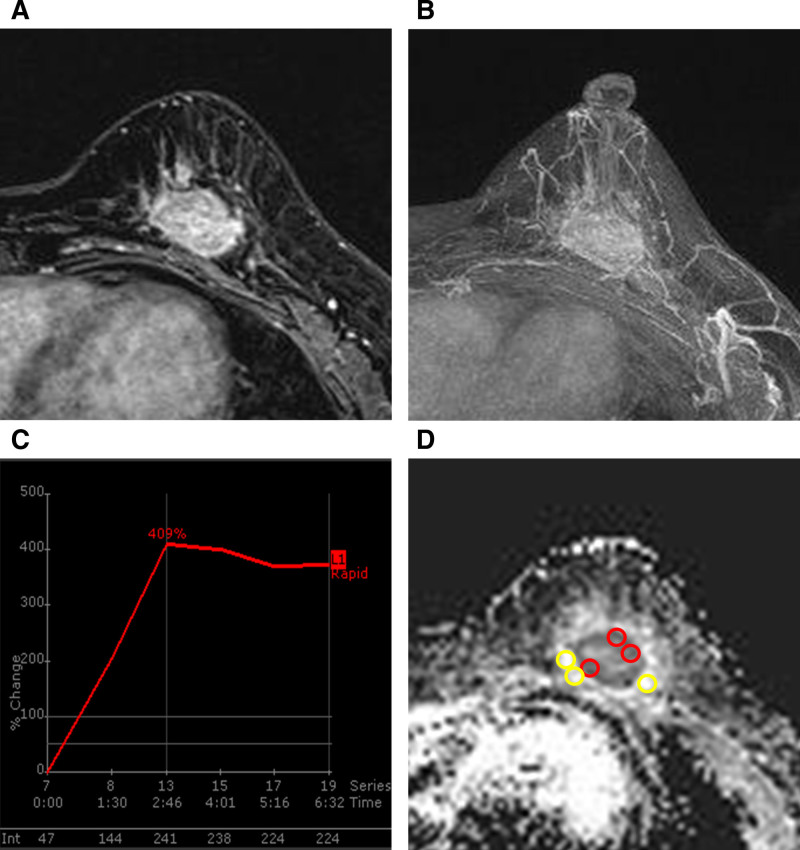
Breast magnetic resonance imaging findings of large cell neuroendocrine carcinoma. (A) The maximum intensity projection (MIP) image shows mildly increased ipsilateral whole-breast vascularity with the adjacent vessel sign. (B) Subtraction image from DCE-MRI revealed a mass in the 6 o’clock position of the left breast with rim internal enhancement. (C) The mass demonstrated a rapid initial enhancement followed by a washout pattern on the kinetic curve. (D) On the ADC map, the minimum mean value among the 3 ROIs measured within the tumor was 745 × 10^−6^ mm²/s, and the maximum value among the 3 ROIs measured in the brightest areas of the peritumoral lesion was 2075 × 10^−6^ mm²/s, resulting in a peritumoral-to-tumoral ratio of 2.79. ADC = apparent diffusion coefficient, DCE-MRI = dynamic contrast-enhanced magnetic resonance imaging, MIP = maximum intensity projection, ROI = region of interest.

A 55-year-old woman visited our hospital because of screening abnormalities. She had a history of taking oral medications and was menopausal. There was no previous personal history or family history of breast cancer. Physical examination revealed a nonfixed palpable mass in the left lower central breast. Mammography revealed a 3-cm, irregularly shaped, hyperdense mass without microcalcification in the lower central quadrant of the left breast (Fig. 1). Breast ultrasound revealed an irregularly shaped, microlobulated margin and hypoechoic solid mass in the left lower breast. The mass had no internal or external microcalcifications, and it lacked posterior features (Fig. 2A). Chest computed tomography showed a microlobulated mass with rim enhancement in the left lower central breast but no enlarged axillary lymph nodes (Fig. 2B). Breast magnetic resonance imaging (MRI) was performed to evaluate tumor extent. The precontrast T2-weighted sequence demonstrated an oval-shaped mass with circumscribed margins and high signal intensity. From the dynamic contrast-enhanced MRI (DCE-MRI), the mass in the left lower central breast showed a rim enhancement pattern with internal enhancement characteristics (Fig. 3A). Around the mass, an adjacent vessel sign and mildly increased ipsilateral whole-breast vascularity were present, and there were no associated features (Fig. 3B). In the time signal intensity curve, a 3-cm mass revealed an initial rapid enhancement and a delayed washout pattern (Fig. 3C).

**Figure 4.**
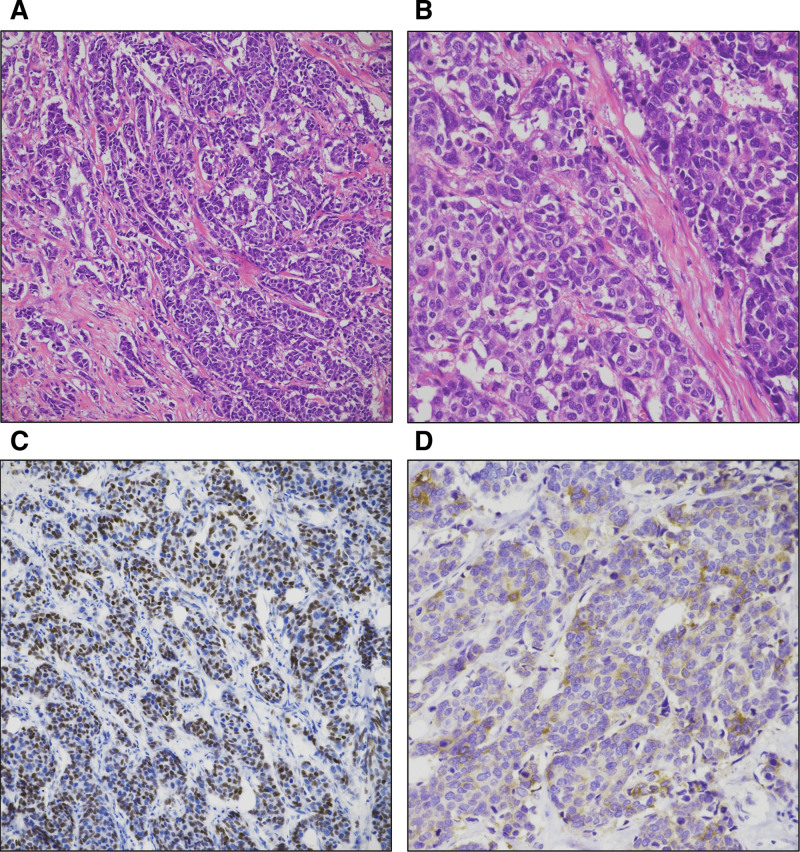
Histopathological and immunohistochemical findings of large cell neuroendocrine carcinoma. (A) Lower power view shows tumor cells with large, polygonal shapes and abundant cytoplasm arranged in solid nests (H&E stain, original magnification: ×100). (B) Higher magnification highlights cellular pleomorphism with occasional nucleoli and abundant mitosis (H&E stain, original magnification: ×200). (C) Immunohistochemical staining for INSM1 demonstrates strong nuclear positivity (original magnification: ×100). (D) Immunohistochemical staining for synaptophysin reveals cytoplasmic positivity (original magnification: ×200). INSM1 = insulinoma-associated protein 1.

To assess the diffusion-weighted image (DWI) characteristics, the radiologists placed regions of interest (ROIs) on apparent diffusion coefficient (ADC) maps (using a 0, 800s/mm^2^ *b*-value) based on the extension of lesions demonstrated by DCE-MRI. On the ADC map, the largest cross-sectional area of the tumor was selected, and 3 circular ROIs (each 4 pixels in size) were carefully placed within the most visually hypointense regions, avoiding the cystic, necrotic, fatty, and hemorrhagic areas. The mean ADC value of each ROI was recorded, and the lowest mean value among the 3 was designated as the tumor ADC, measured at 745 × 10^−6^ mm^2^/s. The maximum peritumoral ADC value was recorded as 2542 × 10^−6^ mm^2^/s, based on 3 ROIs placed on adjacent breast parenchymal tissue where the ADC visually appeared highest. The peritumoral-to-tumoral ADC ratio was calculated as 2.79. The patient underwent a partial mastectomy and sentinel lymph node biopsy. Macroscopically, the tumor measured 3.1 cm in diameter and showed lymphatic invasion; however, the sentinel and axillary lymph nodes had no metastasis. Light microscopy revealed a tumor predominantly composed of large polygonal shapes with abundant cytoplasm arranged in solid nests. Higher magnification highlighted cellular pleomorphism with occasional nucleoli and abundant mitosis. Immunohistochemical analysis revealed diffuse uniform staining for human epidermal growth factor receptor 2, neuroendocrine markers (INSM1 and synaptophysin) (Fig. 4). The tumor cells were negative for estrogen receptor, progesterone receptor, and other neuroendocrine markers (chromogranin and neuron-specific enolase). The patient successfully adhered to the planned complete surgical resection, which was well tolerated without any perioperative complications, and there was no evidence of recurrence or metastasis to any other site. However, the patient did not return for subsequent clinical visits and was lost to follow-up. The sequence of clinical events, imaging findings, and management strategies are detailed in Table 1 for clarity.

**Table 1:** Clinical timeline of the patient with large cell neuroendocrine carcinoma of the breast.

Time point	Event	Findings/notes
Screening (0 months)	Abnormality detected on mammography	3-cm irregular hyperdense mass in the left breast
Presentation (0 months)		Nonfixed palpable breast mass
Diagnostic imaging (0 months)	US, CT, MRI	Irregular hypoechoic mass (US), rim enhancement, rapid washout (MRI), high peritumoral-to-tumoral ADC ratio (2.79)
Diagnosis (0 months)	Core-needle biopsy	Poorly differentiated carcinoma with neuroendocrine features
Surgery (1 month)	Partial mastectomy and sentinel lymph node biopsy	3.1-cm tumor, lymphatic invasion present, no nodal metastasis
Pathology (1 month)	Histopathology and immunohistochemistry	Finally diagnosed with large cell neuroendocrine carcinoma of the breast, large polygonal tumor cells, HER2 positive, INSM1/Syn positive, ER/PR negative
Postoperative outcome (1 month)	After surgery	No evidence of recurrence or metastasis at initial evaluation
Follow-up		The patient did not return for subsequent clinical visits and was lost to follow-up

ADC = apparent diffusion coefficient, CT = computed tomography, ER = estrogen receptor, HER2 = human epidermal growth factor receptor 2, INSM1 = insulinoma-associated protein 1, MRI = magnetic resonance imaging, PR = progesterone receptor, Syn = synaptophysin, US = ultrasound.

## 3. Discussion

LCNECB is a rare malignant tumor that originates in the breast; however, its pathogenesis is not yet clearly understood. Earlier studies have proposed that cancer cells originate from neuroendocrine cells in the breast, although the existence of such cells in normal mammary tissues remains controversial. More recent hypotheses propose 3 possible mechanisms: (1) breast cancer stem cells differentiate into epithelial and neuroendocrine cells, (2) breast epithelial cells acquire neuroendocrine traits during carcinogenesis, and (3) neuroendocrine cells migrate to the breast.^[2,3]^ Despite the differences in their pathways, these mechanisms commonly suggest that neuroendocrine cells emerge during tumorigenesis and not from preexisting cells in the normal mammary tissue. Given these proposed mechanisms of tumorigenesis, identifying noninvasive imaging characteristics suggestive of LCNECB during the initial diagnostic process can therefore facilitate early diagnosis, reduce the need for invasive procedures, and aid in the evaluation of clinical behavior and prognosis.

Radiologically, although the mammographic and sonographic features of LCNECB are generally nonspecific,^[4–8]^ breast MRI may help differentiate LCNECB from other types of NECB or other malignant tumors. DCE-MRI and DWI are useful for characterizing breast lesions by assessing the vascularity, enhancement kinetics, and cellular density, thereby helping differentiate benign from malignant tumors. In this case, breast MRI showed marked hypervascularity of the tumor, with early intense enhancement and rapid washout, accompanied by increased ipsilateral whole-breast vascularity. Although high-grade invasive ductal carcinoma (IDC) or the triple negative subtype of the tumor can demonstrate hypervascular characteristics, particularly in aggressive molecular subtypes, the degree and distribution of vascularity may differ. In aggressive forms of IDC, hypervascularity is typically confined to the tumor itself, reflecting aggressive angiogenesis within the lesion.^[9]^ However, LCNECB has been reported to exhibit marked intratumoral hypervascularity, accompanied by increased ipsilateral whole-breast vascularity, which may reflect a unique vascular microenvironment associated with neuroendocrine differentiation.^[10]^ This is supported by reports of vascular endothelial growth factor-C overexpression and activation of angiogenic pathways in neuroendocrine breast cancers.^[11]^ Furthermore, hypervascularity is a characteristic imaging finding in NETs of other organs, such as the pancreas or gastrointestinal tract, and is often correlated with the presence of a dense capillary network.^[12]^ Although the radiological significance of the increased ipsilateral whole-breast vascularity in LCNECB is not well established, it may reflect a distinct vascular phenotype associated with neuroendocrine differentiation. Nevertheless, further studies using breast MRI are warranted, as considerable overlap in imaging features persists between LCNECB and other aggressive subtypes of breast cancer, and histopathological assessment remains essential for accurate diagnosis and prognostic stratification.

In this case, the ADC value measured on DWI was 0.745 × 10^−3^ mm^2^/s. While there are currently no established data specifically describing the ADC values for LCNECB, previous literature reports have shown that the mean ADC value in IDC is approximately 0.88 × 10^−3^ mm^2^/s.^[13]^ A comparative study of pancreatic neuroendocrine carcinoma and pancreatic ductal adenocarcinoma found that PanNEC had lower ADC values, which were attributed to the higher cellularity of NETs.^[14]^ This characteristic may explain the lower ADC value observed in our case, thus suggesting a more aggressive tumor biology than IDC and providing potential utility in differentiating LCNECB from IDC. In addition, the peritumoral-to-tumoral ADC ratio in our case was 2.79, which is markedly higher than that reported in a previous study on IDC, where it was 1.53 in patients without sentinel lymph node metastasis and 1.62 in those with metastasis.^[15]^ A previous study has demonstrated that higher ratios are significantly associated with adverse prognostic factors such as larger tumor size, higher histologic grade, elevated Ki-67 index, axillary lymph node metastasis, and lymphovascular invasion, and correlate with established prognostic models.^[16]^ This suggests that the ratio may serve as a helpful imaging marker and as a potential surrogate indicator of aggressive tumor biology. In this context, a substantially elevated ratio, as seen in our patient, may reflect a distinctive microenvironment in LCNECB and could ultimately aid in refining diagnostic pathways and prognostic stratification if confirmed in larger cohorts.

## 4. Conclusion

Our case highlights increased ipsilateral whole-breast vascularity, rapid initial enhancement with delayed washout on DCE-MRI features, and low ADC value, and a markedly elevated peritumoral-to-tumoral ADC ratio on DWI features, which aid in differentiating LCNECB from other types of NECB or other malignant tumors and provide prognostic prediction. Although integrating MRI with pathological evaluation is necessary for accurate diagnosis and treatment planning, we hope to demonstrate the value of breast MRI in improving the diagnostic process and guiding clinical decisions for this rare tumor by pointing out these imaging traits.

## Author contributions

**Conceptualization:** Eun Jung Choi.

**Resources:** Kyoung Min Kim.

**Supervision:** Eun Jung Choi.

**Writing – original draft:** Boram Kim.

**Writing – review & editing:** Seoyun Choi, Eun Jung Choi.
